# Describing and explaining consciousness

**DOI:** 10.1093/nc/niad009

**Published:** 2023-04-13

**Authors:** Bjørn Grinde

**Affiliations:** Division of Mental and Physical Health, Norwegian Institute of Public Health, Postboks 222 Skøyen, Oslo 0213, Norway

**Keywords:** evolution, biology, explaining consciousness, feelings, neurobiology

## Abstract

Consciousness is a property of advanced brains and as such a biological feature. Explaining biological features is somewhat different from explaining physical phenomena; in the former case, the key is to first define its functional role (the reason why it was selected) and then to outline the evolutionary trajectory leading to its presence. In the case of consciousness, there are reasonable models for both. Further research is required to substantiate these models, but they offer, arguably, the best explanatory framework.

In a recent special issue of this journal, *Consciousness science and its theories*, Schurger and Graziano (2022) discuss whether current theories of consciousness are simply descriptions rather than explanations. The difference is exemplified by Newton’s law of gravity and Galileo’s depiction of the solar system. The authors consider various attempts at understanding consciousness and conclude that they all constitute descriptions, except for the ‘attention scheme theory’ (Graziano and Kastner 2011).

The examples of Newton and Galileo are to the point when examining physical phenomena; however, in the case of biological phenomena, there is a different explanatory framework. To elucidate features associated with living organisms, one needs to first understand their role in survival and procreation and then to describe a reasonable evolutionary scenario leading to their presence. The former explains why they were selected, and the latter substantiates the explanation by suggesting a plausible historic narrative. As famously pointed out by Dobzhansky (1973), “nothing in biology makes sense except in the light of evolution.” Strangely, the explanatory power of the evolutionary perspective is not discussed by Schurger and Graziano.

I have presented a possible evolutionary explanation for consciousness in detail elsewhere (Grinde 2016, 2018, 2023); here, there is only space for a short summary: The role of the brain is to gather relevant information, make behavioral decisions based on the information, and orchestrate the execution of these decisions. Consciousness represents one of the algorithms that evolved for the purpose of improving the survival value of behavior. Its evolution possibly started some 300 million years ago in the early amniotes. The first step was to convert nonconscious motivators into feelings. Feelings had the advantage of offering a “common currency” that could be used for weighing behavioral options based on their mood value. Positive feelings point toward what is good for the genes, and negative feelings indicate that something should be avoided. The organism senses the mood value associated with present behavior and can anticipate the value associated with various alternatives for future behavior. The information is computed to find the alternative generating the most pleasant outcome. The algorithm offered a flexible and versatile behavioral tool that proved useful in certain situations.

Even in a species such as humans, with presumably the most advanced form of brain, consciousness is only involved in select types of decisions. For example, the regulation of pupil size and the reflexive withdrawal of a finger put on a hot stove are not under conscious control. On the other hand, our advanced cognitive capacity means we can choose not to follow the principle of maximum mood value. We are a special case but that does not negate the suggested evolutionary model of consciousness.

Feelings required an ability to ‘feel’ and thus a form of awareness. Evolution expanded the nascent capacity of awareness by including (select) sensory input; the combination of feelings and sensations constitutes what we refer to as sentience. Later, more advanced capabilities were added, such as self-awareness and the cognitive powers of the more sophisticated animals. Combined with sentience, these add-ons allow for even more fine-tuned, flexible, and accurate behavior.

The abovementioned account needs further supportive evidence, but it offers a straightforward approach to explain what we are dealing with. That said, the evolutionary perspective is not the only way to illuminate consciousness. Other theories and descriptions can add to our understanding. There are many ways to paint a picture of reality that has informative value. My point is that a biological interpretation should be included.

The phenomenon we refer to as consciousness is not necessarily that unique. We are at a similar stage in our attempts to illuminate a vast range of other biological features, such as the capacity of bees to signal the location of a food source, or how salmon navigate the oceans to find back to the river where they were born. In both cases, we have a reasonable grasp of the functional value, we know a bit about the neurological mechanisms involved, and we can postulate an evolutionary scenario leading to their presence. Yet, the models are only rough sketches.

Being able to describe the neurobiology of consciousness in further detail would be a breakthrough, but I agree with Schurger and Graziano that by itself it is primarily a description; a complete understanding requires an evolutionary account. There are neurological models available, such as the global neuronal workspace hypothesis (Mashour et al. 2020). This model supplements the evolutionary model; as discussed elsewhere (Grinde 2018), the two models can mutually guide research in the direction of strengthening (or weakening) both accounts.

In my mind, our capacity to understand the neurobiology of consciousness is roughly at par with our knowledge of other advanced brain functions, such as how minor variations of air pressure outside the ear are transformed into extremely intricate sounds or how the brain can fine-tune the 200 or so muscle movements per second required to speak (MacNeilage 2010). In fact, consciousness may not be the most complex or demanding function (Grinde 2023).

I have presented five biological phenomena that are all amazing: signaling in bees, navigation in salmons, distinguishing sounds, coordinating muscles, and a conscious experience. These phenomena are all products of nervous systems. This means that they are necessarily related in how they are executed; their neurobiological correlates are based on the same repertoire of neurological processes. If we can understand one complex process of the brain in greater detail, it should help us understand the others. We may never be able to offer an exact description of any of these phenomena, but I am sure we will develop more detailed, and better substantiated, models compared to what we have now.

A core question is whether consciousness stands out as radically different from other brain processes. Chalmers (2017), in his description of the “hard problem,” suggests so. The stance is reflected in the observation that the literature on consciousness is characterized by philosophical and metaphysical approaches. However, this observation may be due to the role of consciousness in human life; in a way, it is “all we got” and thus, the one thing we humans really care about. I believe that the hard problem is not a metaphysical obstacle but stems from a deep interest in the capacity to experience the world combined with a lack of knowledge. Obviously, if the obstacle is genuine, then a neurobiological approach will fall short.

The various functions added to the brain by evolution can be compared with apps in a mobile phone (Grinde 2016). In this analogy, consciousness is an app that the brain turns on when assumed to be useful (typically in the morning) and off when no longer required. This app appears to have evolved in the direction of a hostile takeover, akin to the artificial intelligence portrayed in the film The Matrix. Human consciousness, with its accompanying level of free will (Grinde 2022), has reached a point where it can contribute to decisions that are not conducive to survival and procreation, such as not to have children or even kill oneself.

Somewhat surprisingly, the consciousness app does not appear to be particularly advantageous in evolutionary terms, as indicated by the observation that other species with advanced consciousness (such as apes and former hominins) either are extinct or struggle to survive (Grinde 2023). The recent (biological) success of *Homo sapiens* seems to be due to one specific outcome of consciousness, i.e., the capacity it gave us for cultural evolution.

Consciousness is arguably the most interesting phenomenon in biology, and I believe that an explanation requires a biological approach. The chance to improve our insight is best if we investigate the evolutionary process behind and the neurological mechanisms involved. Focusing on the “hard problem” can make the problem harder to solve. The fact that there are a lot of pieces in the puzzle left to be filled in does not negate this stance or make consciousness unique.
